# Human NK Cells: From Surface Receptors to the Therapy of Leukemias and Solid Tumors

**DOI:** 10.3389/fimmu.2014.00087

**Published:** 2014-03-07

**Authors:** Lorenzo Moretta, Gabriella Pietra, Elisa Montaldo, Paola Vacca, Daniela Pende, Michela Falco, Genny Del Zotto, Franco Locatelli, Alessandro Moretta, Maria Cristina Mingari

**Affiliations:** ^1^Istituto Giannina Gaslini, Genova, Italy; ^2^Department of Experimental Medicine and Center of Excellence for Biomedical Research, University of Genova, Genova, Italy; ^3^IRCCS AOU San Martino-IST, Genova, Italy; ^4^Department of Pediatric Hematology and Oncology, IRCCS Ospedale Pediatrico Bambino Gesù, Rome, Italy; ^5^Università di Pavia, Pavia, Italy

**Keywords:** NK cells, killer Ig-like receptors, alloreactive NK cells, activating NK receptors, hematopoietic stem cell transplantation, acute leukemias, tumor microenvironment

## Abstract

Natural Killer (NK) cells are major effector cells of the innate immunity. The discovery, over two decades ago, of major histocompatibility complex-class I-specific inhibitory NK receptors and subsequently of activating receptors, recognizing ligands expressed by tumor or virus-infected cells, paved the way to our understanding of the mechanisms of selective recognition and killing of tumor cells. Although NK cells can efficiently kill tumor cells of different histotypes *in vitro*, their activity may be limited *in vivo* by their inefficient trafficking to tumor lesions and by the inhibition of their function induced by tumor cells themselves and by the tumor microenvironment. On the other hand, the important role of NK cells has been clearly demonstrated in the therapy of high risk leukemias in the haploidentical hematopoietic stem cell (HSC) transplantation setting. NK cells derived from donor HSC kill leukemic cells residual after the conditioning regimen, thus preventing leukemia relapses. In addition, they also kill residual dendritic cells and T lymphocytes, thus preventing both GvH disease and graft rejection.

## Introduction

Natural Killer (NK) cells play a central role in innate immunity as they mediate early defenses against viral infections and, more in general, against pathogens. However, NK cells are also involved in immune surveillance against tumors and prevent dissemination of metastatic tumors (1, 2). The NK effector function against tumors and virus-infected cells is mostly related to their cytolytic activity. In addition, by the secretion of various cytokines and chemokines, NK cells promote inflammatory responses and exert a regulatory control on downstream adaptive immune responses by influencing not only the strength, but also the quality of T cell responses. T helper-1 responses, favored by NK cells, further contribute to anti-tumor and anti-virus defenses. In turn, NK cell function is regulated by cytokines, including IL-15, IL-2, and IL-18 (3) as well as by cell-to-cell interactions involving different cell types primarily dendritic cells (DC) (3–5), macrophages (6), and mesenchymal stromal cells (7, 8). NK cells migrate to inflamed tissue and to secondary lymphoid organs where they can encounter tumor cells and participate to the first line of defense against pathogens. NK cells originate from hematopoietic stem cells (HSC) and undergo maturation primarily in the bone marrow (BM). However, evidence has been accumulated during the past several years that NK precursors at different stages of differentiation are present in tonsils (9), lymph nodes (10), decidua (11), and gut-associated lymphoid tissues (12). In addition, precursors capable of undergoing *in vitro* differentiation toward NK cells were isolated from human thymus over two decades ago (13).

## Inhibitory and Activating NK Receptors: Past and Present

In spite of their functional relevance in defenses against viruses and tumors, NK cells remained mysterious and poorly considered for many years after their discovery (14–16) so that core questions regarding the molecular mechanisms involved in their ability to discriminate between normal and tumor or virus-infected cells remained unanswered. However, starting in early 90s, we began to gain a fair idea on the mechanisms regulating NK cell activation and function. In late 80s, Ljunggren and Kärre had proposed the “missing self hypothesis” (17), based on the observation that NK cells could efficiently kill a murine lymphoma cell line that had lost major histocompatibility complex (MHC)-class I, while the parental MHC-class I^+^ lymphoma cells were resistant to lysis. Thus, it appeared that NK cells could sense MHC-class I molecules, sparing MHC-class I^+^ cells while killing MHC-class I^−^ cells. In addition, a clue that NK cells could sense even allelic differences on hematopoietic target cells was provided by the hybrid resistance phenomenon in which NK cells could reject parental BM graft in F1 hybrid mice (18). Another experiment suggesting that MHC-class I molecules could influence NK cell function was the detection of human NK cell proliferation in mixed lymphocyte culture against stimulating cells from unrelated donors (in the presence of IL-2). In addition, such cultured NK cells could lyse phytohemagglutinin (PHA) blasts isolated from the same stimulating donor (19). Taken together, these data were compatible with the expression, at the NK cell surface, of inhibitory receptors sensing MHC-class I molecules. The discovery of surface molecules expressed by human NK cell subsets that could inhibit the NK cell cytotoxicity upon monoclonal antibody (mAb)-mediated crosslinking (20, 21), was the first step toward the identification of human leukocytes antigen (HLA)-class I-specific inhibitory receptors recognizing allelic forms of HLA-C (22). Remarkably, in parallel, Yokoyama et al. had identified Ly49 molecules as the murine receptors for MHC-class I (23). A number of novel receptors belonging to the same Ig-superfamily of the two HLA-C-specific prototypes (named p58.1 and p58.2) were identified and collectively called killer Ig-like receptors (KIRs). They also recognized allelic forms of HLA-B or -A allotypes (24–27). In addition, activating KIRs were discovered (28) that were similar to the corresponding inhibitory KIRs in the extracellular Ig-domains, but substantially differed in the transmembrane and in the intracytoplasmic portions (29). Both inhibitory and activating KIRs have been shown to play an important role in the cure of high risk leukemias in the haploidentical HSC transplantation setting (see below). Genetic analysis revealed that KIR-encoding genes evolved and diversified rapidly in primates and humans (30). Likewise the HLA loci, KIR sequences were found to be highly polymorphic. KIR genes are organized as a family in the leukocyte receptor complex in chromosome 19 and are inherited as haplotypes. KIR haplotypes exhibit variability in the number and type of genes and in allelic polymorphism of the individual KIR genes, resulting in extensive genetic diversity. On the basis of their gene content, KIR haplotypes have been divided into group A (with a fixed gene pattern mainly including inhibitory KIR) and group B (more variable and including several activating KIR) (31). Other receptors with different HLA-I specificities, including CD94/NKG2A and LIR-1, were discovered and characterized (32, 33). Since inactivation of NK cell function represents a central fail-safe mechanism to prevent killing of normal self HLA-class I^+^ cells, the existence of activating receptors that are triggered upon interaction with normal cells had to be postulated. Experiments aimed at identifying these receptors were successful and three important activating NK receptors named NKp46 (34, 35), NKp44 (36, 37), and NKp30 (38) were discovered and molecularly characterized (39). These molecules, collectively termed natural cytotoxicity receptors (NCRs), were found to play a central role in tumor cell recognition and killing. Additional surface molecules functioning as activating receptors or co-receptors were subsequently identified. Some of these molecules, primarily NKG2D and DNAM-1, were also shown to play an important role in target cell recognition and lysis (40, 41). Remarkably, the known ligands of such receptors are over-expressed or expressed *de novo* upon cell stress, particularly when consequent to tumor transformation or viral infection (40, 42, 43). The fact that NK cell activation may occur only upon interaction with abnormal target cells represents an important checkpoint to control unnecessary NK cell activation (44). In case of NK cell interaction with ligand-positive stressed cells, the latter are protected from lysis because of the engagement of HLA-I-specific inhibitory NK receptors by HLA-I molecules expressed normally, or even upregulated in these cells. On the contrary, virus-infected or tumor cells lack the expression of HLA-I molecules and upregulate the expression of NK activating receptor ligands becoming susceptible to NK cell lysis. The ligands of the main activating NK receptors include the human leukocyte antigen-B-associated transcript 3 (BAT-3) and B7H6 for NKp30 (45, 46), a novel isoform of the mixed-lineage leukemia-5 protein (MLL5) for NKp44 (47), PVR (CD155) and Nectin-2 (CD112) for DNAM-1 (42), and MICA/B and ULBPs for NKG2D (43). Direct identification of such ligands in tumor cells may allow predicting whether a given tumor may be susceptible to NK-mediated killing (see below for details).

## NK Cells and Solid Tumors

Besides specific T lymphocytes, also NK cells are thought to play an important role in cancer immunosurveillance. NK cells are capable of recognizing and killing a wide variety of tumor cells. NK cells are potentially capable of eliminating tumors with reduced or absent MHC-class I expression that evade CD8^+^ T cell-mediated control. Therefore, they are playing a complementary role in anti-tumor activity. Recent studies also suggest that NK cells recognize and kill cancer stem cells (CSCs) (48, 49). Within the tumor mass, CSCs represent a small subpopulation of quiescent, self-renewing, chemo- and radio-resistant cells and hence they are responsible for tumor relapses after cytoreductive therapies.

In clinical studies, the degree of NK-mediated cytotoxic activity has been inversely correlated with cancer incidence in long survey subjects (50). In addition, several studies have provided evidence that, in a variety of different solid tumors, such as lung, gastric, colorectal, and head and neck cancers, the presence of high numbers of tumor-infiltrating NK cells correlates with improved prognosis of cancer patients (51–53). Despite the fact that NK cells represent a potential tool to eliminate tumor cells, NK cell-based immunotherapy has resulted in limited clinical benefit (54). In particular, this holds true in the case of solid tumors, suggesting that mechanisms of resistance at the level of the tumor microenvironment may be prevailing in many cases. This may reflect the limited capacity of adoptively transferred NK cells to traffic to tumor sites (55, 56).

Of note, factors regulating NK cell recruitment into neoplastic tissues are highly influenced by the tumor type, and by the chemokine profile of the tumor microenvironment. Several studies suggested that certain solid malignancies are infiltrated by variable numbers of NK cells. Those include, non-small cell lung cancers (NSCLC), gastrointestinal sarcoma (GIST), colorectal and renal cell carcinoma, and lung metastases (57–59). A recent study suggested that CD56^+^ NK cells could scarcely infiltrate melanomas, hepatocellular carcinomas, breast cancers, and renal cell carcinomas (60). Other studies reported that NK cells in solid tumors are often not located in direct contact with tumor cells but within the stroma (55, 61) and usually functionally anergic.

**Figure 1:**
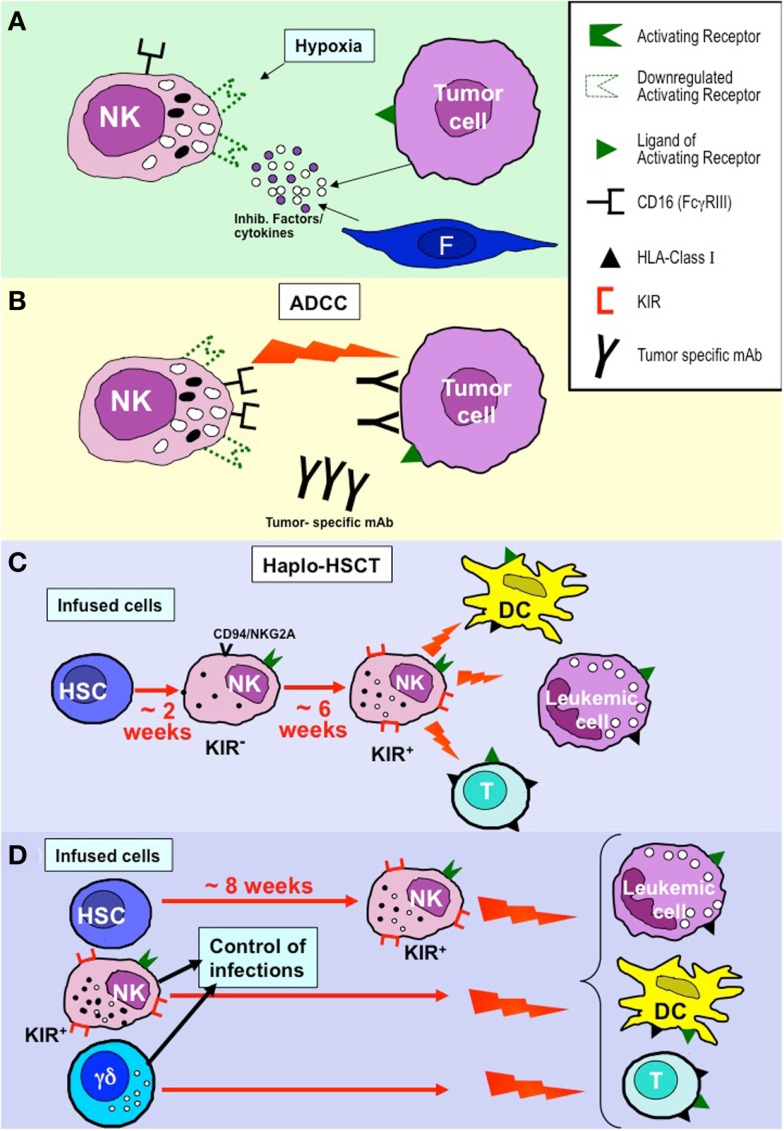
**NK cell-based approaches in the immunotherapy of tumors and leukemias**. **(A)** NK cell function may be greatly hampered by inhibitory factors and/or cytokines produced by tumor cells or cells of the tumor microenvironment (e.g., fibroblasts, F) and by hypoxia that primarily induce down-regulation of activating NK receptors. **(B)** CD16-mediated antibody dependent cytotoxicity (ADCC) appears to be poorly susceptible to the inhibitory tumor microenvironment. This mechanism may contribute to the positive clinical outcome of patients treated with tumor-specific monoclonal antibodies (mAbs). **(C)** In the T-depleted haplo-HSCT, KIR^+^ alloreactive NK cells derived from donor HSC (generated after 6–8 weeks) kill leukemia blasts (inducing GvL), DC (preventing GvHD), and T cells (preventing graft rejection) remaining after the conditioning regimen. **(D)** In haplo-HSCT, early leukemia relapses and severe viral infection may occur during the time interval (6–8 weeks) required for the generation of efficient alloreactive NK cells. The novel approach based on TCR α/β^+^- and B cell-depletion allows the infusion of donor-derived mature alloreactive NK cells and TCR γ/δ^+^ cells together with HSC, thus allowing a better control of leukemia relapses, GvHD, graft rejection, and viral infection/reactivation.

Thus, tumor cells may have developed various escape mechanisms to avoid NK-mediated killing. Hence, the tumor cells themselves or even tumor stromal cells may be actively involved in inhibition of NK cell function. Indeed, the tumor microenvironment may greatly influence NK-mediated defenses by a number of immunosuppressive strategies. Similar to T cells, tumor-infiltrating NK cells may be inhibited in their functional capability (57, 62–64). It has been shown that impaired NK cell function is often associated with down-modulation of activating NK receptors. The molecular mechanisms underlying this down-regulation are only partially understood. In this context, ligand-induced receptor down-regulation may play a relevant role. This may be consequent to receptor blocking by ligand shed from tumor cells or to intercellular transfer (a phenomenon known as trogocytosis) (65, 66). In addition, chronic ligand-induced stimulation of NK cells may account for the down-regulation of activating receptors such as NKG2D (67). Surface molecules expressed by tumor cells could also inhibit NK cell function. For example, MUC16, a glycoprotein expressed on the surface of ovarian cancer cells inhibits synapse formation between tumor cells and NK cells (68). In addition, cytokines or soluble mediators such as TGF-β and PGE2, synthesized either by tumor or by stromal cells down-regulate the surface expression of NKp30, NKp44, and NKG2D and, consequently, NK cell cytotoxicity and cytokine production (69, 70). Furthermore, the enzyme indoleamine 2,3-dioxygenase (IDO) (over-expressed by some tumor cells including melanomas) may also contribute to the establishment of immune tolerance in the tumor microenvironment. In this context, a recent study by our group in melanomas reported that NK cell function may be suppressed by IDO-generated l-kynurenine (a tryptophan-derived toxic metabolite) (71). Finally, also the pro-inflammatory cytokine macrophage migration inhibitory factor (MIF) has been shown to inhibit the NKG2D expression in peripheral blood (PB) NK cells derived from ovarian cancer patients (72) (Figure 1A).

The hypoxic condition in cancer tissues may also contribute to tumor escape from NK cells. In a recent study, we observed that hypoxia can significantly impair both the surface expression and the function of major activating NK receptors involved in tumor recognition, including NKp46, NKp30, NKp44, and NKG2D. Accordingly, the NK-mediated cytotoxicity against tumor cells was sharply decreased under hypoxia conditions (Figure 1A). Interestingly, hypoxia did not affect CD16 (FcγRIII) expression and function. Therefore, NK cells maintained the ability to efficiently kill mAb-coated target cells. These data imply that even at low oxygen tension, targeting of tumors with mAbs may be effective by NK cell-mediated antibody dependent cellular cytotoxicity (ADCC) (73) (Figure 1B).

The described mechanisms of inhibition help to better understand how tumors and their microenvironment can alter the ability of NK cells to elicit an effective anti-tumor response. In view of the immunosuppressive effect exerted by tumor cells at the tumor site, new strategies are required to prevent inhibition of potentially efficient effector mechanisms, for example by blocking the soluble mediators with immunosuppressive activity. Notably, these strategies may be applied to design novel protocols of NK cell-based adoptive immunotherapy to treat solid tumors.

## NK Cells in the Therapy of High Risk Leukemias

Over the past 40 years, allogeneic hematopoietic BM or HSC transplantation from HLA-matched donors has been increasingly used to treat thousands of patients with malignant (primarily leukemias) or non-malignant disorders (e.g., severe combined immunodeficiencies) (74, 75). However, approximately one-third of patients in need of an allograft do not find a compatible donor, including matched-unrelated donors (MUD) and umbilical cord blood (UCB). However, the majority of patients, particularly children or young adults, have a family member identical for one HLA haplotype and mismatched for the other (the so-called haploidentical donor), who could serve as donor of HSC. This, haplo-HSC transplantation offered a promptly available treatment to any patient lacking a matched donor or suitable UCB units (76–78). However, because of the incompatibility at three major HLA loci, it became clear that an extensive T cell depletion was strictly necessary to prevent fatal graft versus host (GvH) reactions (79). T cell-depletion associated to high intensity immunosuppressive/myeloablative conditioning regimens and the use of very large numbers (“megadoses”) of highly purified PB-derived CD34^+^ cells resulted in: (a) the successful engraftment of HSC across the HLA barrier; (b) a very low incidence of grade II–IV acute GvH disease (GvHD), even in the absence of post-transplant prophylactic immune suppression (80–82). However, removal from the graft of mature T cells that, in HLA-matched transplants, are mainly responsible for protection from severe infections resulted in a state of immune deficiency for several months after transplantation. In order to overcome, at least in part, this major disadvantage, the adoptive infusion of T cell lines or clones specific for common life-threatening pathogens, including cytomegalovirus, Epstein–Barr virus, adenovirus, and *Aspergillus*, has been applied successfully in pilot trials (83–85). Another possible consequence of the extensive T cell depletion was a higher rate of leukemia relapses. However, milestone studies in acute myeloid leukemia (AML) adult patients receiving a haplo-HSCT revealed that the graft versus leukemia (GvL) effect was mediated by NK cells generated from donor HSC. This effect was detectable almost exclusively in patients transplanted with donors who had NK cells alloreactive toward recipient cells. These studies clearly indicated that also cells of the innate immunity, such as NK cells, may guarantee a successful clinical outcome in this transplantation setting (81, 82).

The noticeable beneficial effect of alloreactive NK cells, first assessed in adult AML, was subsequently reported in children with high risk acute lymphoid leukemia (ALL) (82, 86, 87). Indeed, the probability of leukemia relapse was very low and the survival rate was at least as good as that of patients receiving a HLA-matched sibling or unrelated donor. Notably, the NK-mediated GvL effect is separated by the occurrence of GvHD, thus clearly indicating that alloreactive NK cells kill leukemia blasts while sparing normal tissues, despite the KIR–HLA-I mismatch. In view of the favorable clinical outcome and the immediate availability of a family haploidentical donor, haplo-HSCT has been included as a valuable option for treating pediatric patients with life-threatening leukemias (88).

In haplo-HSCT, the first wave (occurring after 2–3 weeks) of NK cells derived from donor CD34^+^ HSC cells is composed of CD56^bright^ cells expressing CD94/NKG2A as the only HLA-I-specific receptor. These cells are relatively immature and display low levels of cytolytic activity. The appearance of KIR^+^ NK cells (containing the alloreactive subset) requires four to six additional weeks. Therefore, it is conceivable that an efficient NK-mediated anti-leukemic effect occurs only after this time interval from transplantation (87, 89–91) (Figure 1C).

Given the central role of alloreactive NK cells in preventing leukemia relapses, information on the size of the alloreactive subset in potential donors appeared particularly relevant for optimal donor selection (92). In addition, this information was crucial to assess the generation of this subset in the recipient and its persistence over time. The basic criteria applied for donor selection have been the phenotypic identification of the alloreactive NK cell subset and the assessment of the NK cytotoxicity against leukemia cells (87, 93). Cytofluorimetric analysis, using appropriate combinations of monoclonal antibodies conjugated with different fluorochromes, allowed to identify the alloreactive subset. While only inhibitory KIRs were originally assessed, the more recent availability of mAbs, capable of discriminating between activating and inhibitory KIRs, allowed to extend the analysis to activating KIRs and to better define the size of this subset. This revealed to be particularly important for prevention of leukemia relapses, primarily in donors expressing the activating KIR2DS1, provided that patient’s cells express the ligand of such activating receptor (i.e., HLA-C2 alleles) (87, 93, 94). Other selection criteria have been added that are fundamental particularly in donor–patient pairs in whom no alloreactive NK cells can be found. One is based on KIR genotype analysis, since selection of donors with KIR B haplotypes was associated with significant improvement in disease free survival in adult AML patients. This suggests that activating KIRs, particularly those located in the centromeric portion, play a positive role in GvL (95, 96). In addition, mothers were found to be better donors than fathers (97). By applying all these criteria to donor selection, the survival rate of patients receiving a haplo-HSCT is now over 70% in children with high risk, otherwise fatal, ALL.

As specified above, in haplo-HSCT, the appearance of KIR^+^ NK cells may require 6–8 weeks after donor CD34^+^ cell transplantation. Therefore, their anti-leukemia effect is relatively delayed. In case of rapidly proliferating leukemia blasts and/or of high tumor burden residual after the conditioning regimen, this delay may result in leukemic relapses as well as in impaired control of infections (74). In order to minimize this risk, donor-derived mature alloreactive NK cells, either resting or expanded *in vitro*, can be infused at transplantation or shortly after. A particularly promising approach based on the negative selection of T lymphocytes expressing the αβTCR associated with B cell depletion has recently been applied (98) (Figure 1D). This approach allows the accurate removal of αβ T cells, responsible for the occurrence of GvHD. In addition, in this novel transplantation setting, it is possible not only to transfer to the recipient high numbers of CD34^+^ cells, but also mature NK cells and γδ T cells. Thus, mature, alloreactive NK cells can promptly exert their anti-leukemia activity and prevent GvHD. A similar effect can be mediated by γδ T cells in virtue of their ability to kill leukemia blasts (which express ligands recognized by NK cells and/or γδ T cells). In addition, both cell types can control viral infections or reactivation that may represent life-threatening complications in these patients (99). Additional donor selection criteria can be based also on the higher proportion of NK and γδ T cells in their PB. Preliminary data are particularly encouraging even against pediatric AML that were not cured efficiently by the conventional haplo-HSCT approach upon infusion of CD34^+^ cells (Locatelli et al. study in progress). An additional particularly promising approach resides in NK cell manipulation using anti-KIR mAbs (100). These mAbs, now studied in phase II clinical trials in patients with multiple myeloma or AML, can stably block KIRs and allow NK-mediated killing of autologous or HLA-matched tumor or leukemia cells, thus conferring alloreactivity to any KIR^+^ NK cell.

In conclusion, the discovery of NK cell receptors and of the NK alloreactivity represented a true revolution in allo-HSCT and in the cure of otherwise fatal leukemias.

## Conflict of Interest Statement

Alessandro Moretta is a founder and shareholder of Innate-Pharma (Marseille, France). The remaining authors declare no conflicts of interest.
